# Microaceticoccus formicicus gen. nov., sp. nov., an ammonia-tolerant formate-utilizing bacterium originating from a biogas process

**DOI:** 10.1099/ijsem.0.006773

**Published:** 2025-05-08

**Authors:** George Cheng, Anna Schnürer, Maria Westerholm

**Affiliations:** 1 Department of Molecular Sciences, Swedish University of Agricultural Sciences, Uppsala, Sweden

**Keywords:** ammonia tolerant, anaerobic digestion, formate metabolism, *Microaceticoccus*, *Peptoniphilaceae*

## Abstract

A strictly anaerobic bacterial strain, designated as AMB_02^T^, was isolated from a propionate enrichment culture obtained from a high-ammonia biogas digester. The cells were anaerobic and coccoid (0.5 µm), often appearing as diplococci or in a short chain of three to four cells. Growth was observed between 20 and 45 °C (optimum at 37–39 °C), with an initial pH of 6.5–9.0 (optimum pH 8.0–8.5), and the species tolerated up to 0.8 M NH_4_Cl and 0.5 M NaCl. The major cellular fatty acids were C_16 : 0_ (31.6%), C_14 : 0_ (14.6%) and C_18 : 0_ (13.3%). AMB_02^T^ grew with formate, carbohydrates and aa, including asparagine, histidine, tryptone and tryptophan. Acetate was the major product formed. Phylogenetic analysis based on 16S rRNA gene sequences showed that strain AMB_02^T^ was most closely related to the species *Citroniella saccharovorans* (92.5%). The genome of strain AMB_02^T^ was 2.5 Mb in length with a G+C content of 34.8 mol%, and 2,354 protein-coding genes were predicted. Furthermore, genes coding for the reductive glycine pathway potentially used for formate metabolism were identified. Comparative genomic analysis of AMB_02^T^ revealed the closest similarity to *C. saccharovorans* [21.2% digital DNA–DNA hybridization (dDDH) and 77.4% average nt identity (ANI)] and to *Parvimonas micra* (*2*4.4% dDDH and 76.9% ANI). Based on the phenotypic characteristics and phylogenetic analyses, AMB_02^T^ is regarded as a novel genus, *Microaceticoccus*, within the family *Peptoniphilaceae* for which the species name *Microaceticoccus formicicus* is proposed. The type strain is AMB_02^T^=DSM 110248^T^=JCM 39108^T^.

## Introduction

The biogas process generates renewable energy and a nutrient-rich residue that can replace fossil-based mineral fertilizers and represents an important function in the fight against climate change and societal challenges. Additionally, the anaerobic digestion process can be utilized in the production of green biocommodities from different organic waste streams [1]. Biogas is produced from the anaerobic degradation of various organic waste materials, and the success of the degradation process depends on the community of diverse anaerobic micro-organisms. These micro-organisms conduct a series of complex and interlinked reactions often divided into the main steps hydrolysis, acidogenesis, acetogenesis and methanogenesis [2]. These interactions can be indicated from the functional gene content revealed via culture-independent omics of complex microbial communities, but there is a need to isolate species to unveil their capabilities and accurately determine their role in the biogas system.

Several of the waste materials commonly used as feedstock in biogas systems, such as food waste and animal manure, have a high content of proteins. These materials possess a high biogas potential, and the anaerobic degradation process generates a solid residue of high quality as fertilizer [3,4]. However, the degradation of proteins forms ammonia, which at high levels can lead to the inhibition of many microbial species [5]. The inhibition caused by ammonia is a common process disruption in biogas systems that decrease biogas production [6], while leading to an increase in intermediary compounds, such as the volatile fatty acids (VFAs) acetate and propionate. The accumulation of these VFAs is highly undesirable since it reduces the methane yield and increases the risk for process breakdown [7]. Fortunately, with microbial adaptation and process management, the biogas processes can continue to function at high levels of ammonia [5]. This requires that the process is operated in a manner that supports ammonia-tolerant species. However, to guide the plant operators on how to optimally operate biogas systems utilizing proteinaceous waste, there is a need to increase the knowledge of ammonia-tolerant microbial populations that play key roles in the degradation processes. In addition, the identification and characterization of novel species from the anaerobic degradation systems can be utilized for the development of future biotechnology.

In this article, we characterize the ammonia-tolerant bacterial strain AMB_02^T^ originating from a high-ammonia biogas process, for which we propose the name *Microaceticoccus formicicus*, with AMB_02^T^ as a type strain. Strain AMB_02^T^ demonstrated its ability to grow on aa, carbohydrates and formate and harbour several genes related to respective degradation pathways. The isolated species presented in this study is a member of the family *Peptoniphilaceae*, in which other members have been frequently isolated from anaerobic environments originating from human clinical specimens, gut systems and biogas processes, partaking in roles responsible for protein degradation [8,10]. Members of this family are Gram-stain-positive, strictly anaerobic cocci that produce butyrate, acetate and lactate from peptone and aa metabolism [8].

## Isolation

The strain AMB_02^T^ was recovered from an enrichment culture obtained from an anaerobic enrichment reactor. In short, the enrichment reactor had been inoculated with sludge from a lab-scale, mesophilic high-ammonia biogas reactor (Uppsala, Sweden, May 2019, 5.4 g NH_4_^+^-N l^−1^, 0.6–0.9 g NH_3_ l^−1^). The enrichment reactor operated at 37 °C and was continuously fed a bicarbonate-buffered medium, containing yeast extract (0.2 g l^−1^), sodium propionate (0.1 M, 9.6 g l^−1^) and NH_4_Cl (0.3 M, 16 g l^−1^) as described previously [11]. The reactor had been operated for 6 months under constant conditions at the point of sampling. In an effort to isolate propionate-oxidizing bacteria, agar shakes with added pyruvate (10 mM, 0.88 g l^−1^) as the carbon source were prepared as described previously [12]. Pyruvate is a common substrate used by syntrophic microorganisms in pure culture cultivation [13] and was therefore used in the present study. Greyish, spherical colonies were transferred via syringe to anaerobic bottles containing bicarbonate-buffered basal medium (Table S1, available in the online Supplementary Material) and pyruvate as described in Westerholm *et al*. [12]. Strain AMB_02^T^ was cultivated in the dark, sans shaking at 37 °C with an initial pH of 7.3, unless stated otherwise.

## Phylogeny and sequencing and genomic features

**Fig. 1.**
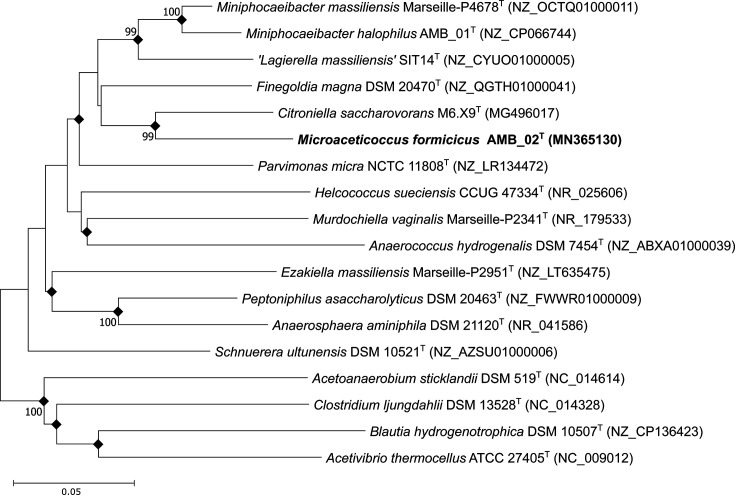
Neighbour-joining phylogenetic tree based on 16S rRNA gene sequences of strain AMB_02T and closely related species. Black diamonds indicate branches that were conserved and recovered in maximum-likelihood and maximum-parsimony trees (Fig. S1). Bootstrap values (based on 1,000 iterations) are shown at the nodes, and only values >70% are shown. Scale bar represents 0.05 substitutions per nt position.

For phylogenetic analysis, DNA was extracted, and the partial 16S rRNA gene was sequenced using the universal bacterial primers 27F/1492R as previously performed [11]. The near complete 16S rRNA gene (1,420 bp) was submitted to the NCBI blastn with the options to exclude models and uncultured/environmental sample sequences to identify closely related species based on the 16S rRNA gene. Extracted sequences of the related strains (Table S3) were aligned using MAFFT [14] (10 retree and 1,000 global pair iterations), and the phylogenetic tree was constructed using neighbour-joining [15], maximum-likelihood [16] and maximum-parsimony [17] methods in mega X [18]. Each tree was evaluated with a bootstrap analysis of 1,000 iterations [19]. The 16S rRNA gene analysis of AMB_02^T^ positioned this species within the family *Peptoniphilaceae* (Figs 1 and S1). The closest relatives, according to the NCBI blastn result of the 16S rRNA gene sequence similarity, were *Citroniella saccharovorans* (92.52% [20]), *Finegoldia magna* (90.21% [21]), *Miniphocaeibacter halophilus* (88.63% [22]) and *Parvimonas micra* (88.60% [23]).

For whole-genome sequencing, the NucleoBond kit (Macharey-Nagel) was used for DNA extraction according to the protocol described in Sun *et al*. [24], with modifications replacing steps 1–6 with centrifugation of 80 ml of culture with an OD_600_ (*λ*=600 nm) of 0.2. The DNA was cleaned with AMPure XP beads (Beckman Coulter) and eluted with 50 µl of sterile water. DNA concentration was quantified with Qubit dsHS DNA assay (Thermo Fisher Scientific), and DNA size was visualized by agarose gel electrophoresis. PCR adapters from Oxford Nanopore Technology were ligated to the DNA strains using the LSK-109 Ligation Sequencing kit. Long-read sequencing was performed using a MinION device (Oxford Nanopore Technologies) until no sequencing activity was observed (~72 h), using a R9.4.1 flow cell (FLO-MIN106) and the MinKNOW software with active channel selection enabled and base calling deactivated. A ‘flow cell-refuel’ step was performed after ~18–20 h of runtime, by adding 75 µl of a 1 : 1 water-SQB buffer mixture (LSK109 kit) to the flow cell via the SpotON port.

Base calling and demultiplexing of raw sequencing data were completed with guppy (v4.0.15-1 –bb42e40) and then filtered with filtlong (v0.2.0) [25]. The genome assembly was performed with flye (v2.8) [26], followed by polishing with racon (v1.4.13) and medaka (v1.0.3) [27]. Minimap2 (v2.17) was used for read mapping to polish the assembly. Genome annotation was conducted with PROKKA (v1.14.6 [28]). The assembly was submitted to the NCBI database with the accession number CP143259.1. The genome of AMB_02^T^ comprised 2,510,182 bp, with a total of 2,411 predicted genes, of which 2,354 (97.6%) were protein coding. The DNA G+C content was 34.8 mol%. Three copies of the 16S rRNA gene, all showing 100% pairwise identity, were identified along with 46 tRNA genes (Table 1).

**Table 1.** Genomic features of AMB_02^T^ and the closest relatives 1, AMB_02^T^; 2, *C. saccharovorans* DSM 29873^T^ [20]; 3, *F. magna* DSM 20470^T^ [21]; 4, *M. halophilus* DSM 110247^T^ [22]; and 5, *P. micra* ATCC 33270^T^ [23]

	1	2	3	4	5
Genome size (bp)	2,510,182	1,953,712	2,020,332	2,394,777	1,733,605
DNA G+C content (mol %)	34.8	30.2	31.8	29.0	29.0
Number of total genes	2,411	2,804	1,925	2,391	1,615
Protein-coding genes	2,354	1,921	1,856	2,287	1,539
5S rRNA gene	4	2	2	4	4
16S rRNA gene	3	2	1	4	3
23S rRNA gene	3	5	1	4	3
tRNA	46	37	30	45	43

**Fig. 2.**
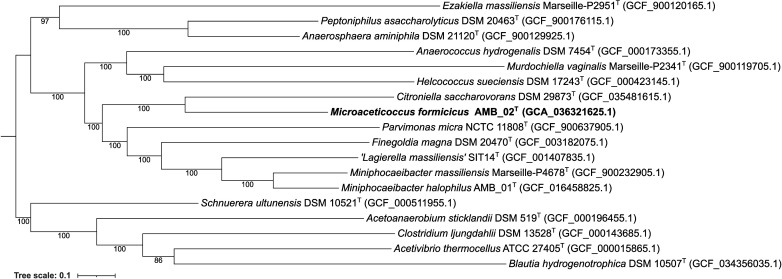
Reconstructed species tree generated using OrthoFinder2, based on whole-genome sequences and 199 single-copy orthologous genes. The tree includes the closest known relatives of strain AMB_02T within the family *Peptoniphilaceae*. Bootstrap values from 1,000 replicates are indicated at branch nodes. Scale bar represents 0.1 substitutions per nt position.

To determine the taxonomy of AMB_02^T^, the approximate taxonomic classification was completed using GTDB-Tk (v2.3.2) [29] method of concatenating multiple sequence alignment of 120 marker genes. Reference genome sequences of the representatives within the family *Peptoniphilaceae* from the NCBI were used and selected using a custom script for dereplication (v0.3.2, https://github.com/rrwick/Assembly-Dereplicator). GToTree (v1.6.34) was used to reconstruct an initial phylogenetic tree to determine the tentative placement of strain AMB_02^T^, based on 119 marker genes selected to cover the phylum *Bacillota* (formerly *Firmicutes*) (Fig. S2). A whole-genome phylogenetic species tree was subsequently reconstructed to further explore the taxonomic placement of the AMB_02^T^ in relation to its close relatives using OrthoFinder2 [30], with 199 single-copy orthologous genes, applying STAG [31] for species tree inference and STRIDE [32] for inferencing root position from gene duplication events. Additional options were used for OrthoFinder2: msa option selected for multiple sequence alignment and IQ-TREE selected for tree inference. Visualization and annotation of the species tree were completed with Figtree (v1.4.4) [33]. The phylogenetic analyses based on the genome sequence revealed that the closest relatives of the novel isolate were *C. saccharovorans*, *P. micra* and *F. magna* (Figs 2 and S2).

The digital DNA–DNA hybridization (dDDH), the average nt identity (ANI) and the average aa identity (AAI) analyses between the genome of strain AMB_02^T^ and available genomes of closely related species were calculated using Genome-to-Genome Distance Calculator 3.0 [34] and the online tool for ANI and AAI calculation from Kostas Lab [35], respectively. The dDDH values between AMB_02 ^T^ and *C. saccharovorans*, *P. micra*, *M. halophilus* and *F. magna* were 21.2%, 24.5%, 20.6%, and 25.4%, respectively. The ANI values of AMB_02^T^ to *C. saccharovorans, P. micra, M. halophilus* and *F. magna* were 77.4%, 76.9%, 75.7% and 74.8%, and the AAI values of AMB_02 ^T^ to *C. saccharovorans*, *P. micra*, *M. halophilus* and *F. magna* were 53.8%, 48.9%, 50.2% and 48.2%, respectively. Consequently, strain AMB_02^T^ was only distantly related to other representatives of the *Peptoniphilaceae* family based on the dDDH, ANI and AAI values, which were below the suggested cut-off values (<70% dDDH, <96% ANI and <95% AAI) to assign the strains to the same genomic species [36,37]. Furthermore, several suggestions for genus delineation cut-offs have been suggested, AAI ranging 55–80% [36,38, 39]. Hence, the AAI value between AMB_02^T^ and the closely related species of 53.8% was also below the suggested cut-offs for the genus delineation.

Analysis of the genome predicted the presence of common biosynthesis pathways involving the synthesis of aa, fatty acids and vitamins. For energy metabolism, genes encoding for the V/A type H^+^/Na^+^-transporting ATPase (*ntp*A-D, K), NADH-quinone oxidoreductase (*nuo*E, F) and NADP^+^-reducing hydrogenase subunits (*Hnd*A, C) were found. Complete sets of genes for glycolysis, pyruvate oxidation and C10-C20 isoprenoid biosynthesis module were found in the genome. Since strain AMB_02^T^ is an acetate-producing bacterium isolated from a biogas system, there was interest in determining if this isolate was an acetogen. The Wood–Ljungdahl pathway (WLP) is utilized by acetogens as the main method for energy conservation and acetyl-CoA synthesis yielding acetate as a product [40]. The analysis of the predicted genes of AMB_02^T^ revealed the presence of genes typically involved in the initial steps of the WLP, including formate-tetrahydrofolate ligase (*fhs*), methenyltetrahydrofolate cyclohydrolase (*fol*D), methylenetetrahydrofolate reductase (*met*F) and formate dehydrogenase (*fdh*A). Additionally, phosphate acetyltransferase (*pta*) and acetate kinase (*ack*A) were predicted. However, the rest of the genes encoding enzymes for the WLP was not found, including the carbon monoxide dehydrogenase/acetyl-CoA synthase complex (*cdh*D, E*/acs*A-E), indicating that AMB_02^T^ is not an acetogen. The reductive glycine pathway (rGlyP) has been suggested to be used for carbon assimilation and acetate production. This pathway integrates the first four steps of the WLP with the glycine synthase system, which is part of the rGlyP [41]. Expanding the search, strain AMB_02^T^ possesses genes encoding for the glycine reductase complex (*grd*A-E) and glycine dehydrogenase (*gcv*PA, PB) and thioredoxin reductase (*trx*A-B). The rGlyP can be used to assimilate formate and CO_2_, particularly relevant since this strain could convert formate to acetate. This pathway has also been suggested to be utilized by syntrophic acetate-oxidizing bacteria [42]. However, this capability was not observed in strain AMB_02^T^. Additionally, no antibiotic resistance genes were found, following the annotation analysis method from Sun *et al*. [43], using ABRicate (v0.8.13) combined with multiple databases, NCBI, CARD, ARG-ANNOT and ResFinder.

## Morphology and growth characteristics

The cell morphology of AMB_02^T^ was determined by using phase-contrast microscopy (DMI 4000 microscope, Leica) and capturing images with DFC360 FX (C-mount adapter: 0.7×, Leica) monochrome fluorescence camera. Gram reaction was completed using conventional staining as described in Halebian *et al.* [44] and by KOH (3%) test under anaerobic conditions according to a previous description [44, 45], with the modification that the cells were pelleted by centrifugation of liquid medium and used instead of a colony from solid medium. The cells of AMB_02^T^ were cocci with a diameter of about 0.5 µm (Fig. S3), and, unlike other members of the *Peptoniphilaceae* family, AMB_02^T^ demonstrated motility [8]. According to both staining and the KOH test, AMB_02^T^ was Gram-stain-negative, which is also contrary to many members of the *Peptoniphilaceae*, most of which stain Gram-positive [8].

Anaerobic cultivation of AMB_02^T^ was performed in anaerobic serum bottles (118 ml, Nordic pack, Sweden) sealed with rubber stoppers (Rubber By, Netherlands). Cultivation for the determination of substrate utilization and pH and temperature ranges and tolerance of ammonium chloride and NaCl was conducted in 20 ml bicarbonate-buffered media (BM), prepared as previously described in Westerholm *et al.* [12] (Table S1). In addition, to compare substrate utilization between AMB_02^T^ and its closest relative, *C. saccharovorans*, medium 2 was used [20,46] (Table S2), as *C. saccharovorans* was unable to grow on BM. In the substrate tests for both AMB_02^T^ and its closest relative, *C. saccharovorans* on medium 2, glucose, maltose and cellobiose were omitted from medium 2 to avoid growth on sugars. For other added substrates, a concentration of 10 mM was used in BM and in medium 2, if not stated otherwise. A control without substrate (or without electron acceptor) was prepared simultaneously. Confirmation of growth on the different substrates was assessed by visual examination of turbidity and by analyses of degradation products by HPLC. The level of product formation from growth on yeast extract as the sole substrate was subtracted from the results. When grown on BM, the isolate produced acetate as a main product (0.4–1.3 g l^–1^) during growth on formate, various monosaccharides and aa (asparagine, betaine, casamino acids, galactose, glucose, fructose, histidine, serine and tryptone), while lower acetate levels (0.1–0.3 g l^–1^) were detected from cysteine, maltose, ribose and tryptophan. Low levels (~0.2 g l^–1^) of butyrate were formed from cysteine and ethanol. In BM, the isolate did not utilize acetate (25 mM, 1.5 g l^–1^), acetoin, arabinose, benzoic acid, 1-butanol, 2,3-butanediol, cellobiose, citrate, dimethylamine, ethanolamine, glycerol, ethylene glycol (5 mM, 0.3 g l^–1^), fumaric acid, lactose, lactate, leucine, malic acid, mannitol, mannose, methanol, methionine, methylamine, phenylalanine, proline, 1,2-propanediol, 2-propanol, pyruvate, raffinose, salicin, sorbitol, sucrose, syringate (2 mM, 0.39 g l^–1^), vanillic acid (3 mM, 0.50 g l^–1^) and xylose (2 mM, 0.30 g l^–1^) (Tables 2 and S4). To explore syntrophic acid oxidation ability, AMB_02^T^ was also inoculated in BM containing 50 mM acetate with *Methanoculleus bourgensis* sp. MAB1, a hydrogenotrophic ammonia-tolerant methanogen, commonly found as a partner to ammonia-tolerant syntrophic acetate-oxidizing bacteria [47,48], or 50 mM propionate with syntrophic acetate-degrading culture (co_mix_ described elsewhere [47]). However, no acetate nor propionate degradation was obtained by these cultures, contradicting syntrophic propionate/acetate-oxidizing capacities of AMB_02^T^. After the cultivating period, samples were collected for HPLC analysis to quantify degradation products. When grown on medium 2, AMB_02^T^ utilized asparagine, betaine, cellobiose, cysteine, galactose, glucose, formate, fructose, histidine, leucine, maltose, mannose, pyruvate, serine, tryptone and tryptophan. Compared to its closest relative, *C. saccharovorans*, both species utilized the aa histidine, leucine, and serine as well as the monosaccharide glucose (Table 2). For both isolates, acetate (0.12–1.7 g l^–1^) was the main product. The discrepancy in substrate utilization by AMB_02^T^ between BM and medium 2 may be attributed to the difference in nutrient content (e.g. casitone and yeast extract) that could alter the fermentation capacity.

**Table 2.** Substrate growth characteristics* for AMB_02^T^ in BM and modified medium 2† and *C. saccharovorans* DSM 29873^T^ in modified medium 2† Legend; ++, Strong growth; +, weak growth; −, no reaction.

Substrate	AMB_02^T^ in BM	AMB_02^T^ in medium 2	*C. saccharovorans* in medium 2
Asparagine	++	++	−
Betaine	++	++	−
Casamino acid	++	−	−
Cellobiose	−	+	−
l-Cysteine	+	++	−
d-Galactose	++	++	−
d-Glucose	++	++	+
Formate	++	++	−
Fructose	++	++	−
Histidine	++	++	+
Leucine	−	++	+
Maltose	+	++	−
Mannose	−	++	−
Proline	−	−	−
Pyruvate	−	++	−
Ribose	+	−	+
Serine	++	++	++
Tryptone	++	++	−
Tryptophan	+	++	−

*A complete table of substrate growth characteristics of AMB_02T when grown in BM is available in Table S4.

†In modified medium 2, sugars are omitted from the medium as described in the section Morphology and growth characteristics.

The pH and temperature ranges were determined in BM at intervals of 0.5 pH units and 2–3 °C. To help mitigate pH drift during pH measurements, the isolate was inoculated in BM with the following modifications: KH_2_PO_4_ or Na_2_HPO_4_ was increased to 40 g l^–1^ for inoculation at low or high pH, respectively. Minor pH adjustments were achieved utilizing HCl or Na_2_CO_3_, while flushing with N_2_/CO_2_ at 25 °C [49]. The ammonium chloride and NaCl tolerances were determined with consecutive transfer with increasing concentrations, at intervals of 0.05 M NH_4_Cl and 0.05 M NaCl at pH 7.3, using glucose as substrate. The optimal conditions for growth were assessed by continuous measurements of OD_600_ during growth. The OD from uninoculated controls was used as a reference. The optimal growth of AMB_02^T^ was obtained at pH 8.0–8.5 (Fig. S4), weak growth was observed at pH 6.5 and 9.0 and the strain did not grow at pH 6.0 and 9.5. Compared to the taxonomically closest relatives, AMB_02^T^ had a comparably higher pH optimum (Table 3 and Fig. S4), reflecting the environmental condition of its isolation source, i.e. a high-ammonia biogas system [47]. On the other hand, *C. saccharovorans* had a pH optimum ranging from 6.5 to 7.3, which notably overlaps with the pH range of human faeces (6.0–7.2), the isolate source of that species [50]. The temperature range for optimum growth was 37–39 °C, weak growth was observed between 20 and 45 °C, similar to the close relatives (Table 3), and the ranges between 15–20 and 45–50 °C did not support growth. AMB_02^T^ tolerated up to 0.8 M NH_4_Cl and 0.5 M NaCl. The ranges of the pH and temperature that supported the growth of strain AMB_02 ^T^ were similar to the ranges of the closest relative *C. saccharovorans* (Table 3).

**Table 3.** Compilation of the characteristic features of strain AMB_02^T^ and the closest relatives.Characteristics for the closest relatives were retrieved from their original presentation. 1, AMB_02^T^; 2, *C. saccharovorans* DSM 29873^T^ [20]; 3, *F. magna* DSM 20470^T^ [21]; 4, *M. halophilus* DSM 110247^T^ [22]; 5, *P. micra* ATCC 33270^T^ [23]

	1	2	3	4	5
Cell size (µm)	0.5	–	0.8–1.6	0.5	0.3–0.7
Cell morphology	Cocci	Cocci	Cocci	Cocci	Cocci
Temperature for growth (°C)					
Optimum	37–39	30	na	37–42	na
Range	20–45	25–37	20–40	20–45	na
pH for growth					
Optimum	8.0–8.5	6.5–7.3	7.5	7.5–8.0	na
Range	6.5–9.0	6.5–9.0	7.0–9.0	5.5–8.5	na
NaCl tolerance	0.5 M	1 M	na	0.6 M	na
Ammonia tolerance	0.8 M	na	na	0.8 5M	na
Main products	Acetate	Acetate; methyl succinate	Acetate	Acetate	Acetate
Source of isolate	High-ammonia biogas system	Human faeces	Human skin	High-ammonia biogas system	Human gut

na, not applicable.

## Chemotaxonomy

AMB_02^T^ and its closest relative, *C. saccharovorans*, were grown on 700 ml of medium 2, and 500 mg of wet biomass was collected and suspended in isopropanol and sent for commercial analysis of cellular fatty acid composition performed by the DSMZ Identification Service (Leibniz-Institute DSMZ-Deutsche Sammlung von Mikroorganismen und Zellkulturen GmbH, Braunschweig, Germany) after conversion into fatty acid methyl esters by saponification, methylation and extraction using minor modifications of previous descriptions. The fatty acid methyl ester mixtures were separated by GC and detected by a flame ionization detector using Sherlock Microbial Identification System (MIS) (MIDI TSBA40, Microbial ID, Newark, DE 19711, USA). Peaks were automatically integrated, and fatty acid names and percentages were calculated by the MIS Standard Software. Three major fatty acids of strain AMB_02^T^ were C_16 : 0_ (31.6%), C_14 : 0_ (14.6%) and C_18 : 0_ (13.3%), while *C. saccharovorans* had C_16 : 0_ dimethylacetal (DMA) (29.8%) and C_16 : 1_ cis 7 DMA (16.7%) as major fatty acids (Table 4).

**Table 4.** Cellular fatty acid patterns of strain AMB_02 ^T^ (1) and closely related species *C. saccharovorans* DSM 29873^T^ (2) grown on medium 2.Bold face values are major fatty acid groups greater than 10%

Fatty acid	1	2
C_9 : 0_	–	–
C_10 : 0_	–	–
C_12 : 0_	3.3	0.6
C_13 : 0_	–	–
C_14:0_ ALDE	–	_
C_14 : 0_ alcohol	–	_
C_14 : 1_ cis 5	–	_
C_15:0_ ALDE	–	_
C_14:0_ iso	4.3	_
C_14 : 0_	**14.6**	4.6
C_14 : 0_ DMA	–	9.9
C_15:0_ iso	4.5	–
C_16:1_ cis 7 ALDE/C_16:0_ cis 9 ALDE	–	5.7
C_15 : 0_	0.7	–
C_15:0_ anteiso	7.9	–
C_15 : 0_ DMA	–	1.6
C_14 : 0_ 3OH	1.9	–
C_16:0_ iso	9.3	
C_16 : 0_	**31.6**	1.5
C_16:0_ ALDE	_	6
C_16:1_ cis 7	_	2.2
C_16:1_ cis 9	_	1
C_16:1_ cis 7 DMA	_	**16.7**
C_16:1_ cis 9 DMA	_	5.1
C_16 : 0_ DMA	_	**29.8**
C_17:1_ cis 9	_	0.8
C_17:1_ cis 9 DMA	_	0.2
C_17:1_ cis 11 DMA	_	0.4
C_17:0_ iso	2.3	–
C_17:0_ DAM	–	0.5
C_17:0_ anteiso	2.4	–
C_17 : 0_	1	–
C_18:1_ cis 9 DMA	–	3.8
C_18:1_ cis 11 DMA	–	3.5
C_18 : 0_ DMA	–	1.2
C_18 : 0_ iso	1.2	–
C_18 : 0_	**13.3**	–
C_20 : 0_	1.7	–

ALDE, aldehyde.

Considering the morphologic, physiologic, metabolic, chemotaxonomic, phylogenetic and genomic properties of strain AMB_02^T^, it is concluded that it represents a new genus and species within the family *Peptoniphilaceae*, for which the name *Microaceticoccus formicicus* gen. nov., sp. nov. is proposed.

## Description of *Microaceticoccus* gen. nov.

*Microaceticoccus* [Mi.cro.a.ce.ti.coc'cus. Gr. masc. adj. *mikros*, small; L. neut. n. *acetum*, vinegar; N.L. masc. n. coccus (from Gr. masc. n. *kokkos*, a grain), a coccus; N.L. masc. n. *Microaceticoccus*, a small acetic acid forming coccus].

Cells are Gram-stain-negative, strictly anaerobic, cocci shaped and motile with about 0.5 µm in diameter. The colony morphology is round and grey. The cultures are unpigmented. The major fatty acids are C_16 : 0_, C_14 : 0_ and C_18 : 0_. Isoprenoid synthesis modules were detected. The genomic DNA G+C content is about 34.8%. This genus belongs to the *Peptoniphilaceae* family. The type species is *Microaceticoccus formicicus*.

## Description of *Microaceticoccus formicicus* sp. nov.

*Microaceticoccus formicicus* (for.mi'ci.cus. N.L. neut. n. *acidum formicicum*, formic acid; N.L. masc. adj. *formicicus*, pertaining to formic acid).

Displays the following characteristics in addition to those given in the genus description: Growth occurs between 20 and 45 °C (optimum 37–39 °C) and pH range between 6.5 and 9.0 (optimum 8.0–8.5). Tolerates up to 0.5 M NaCl with optimum growth at 9.3 g l^−1^ (0.16 M) NaCl and up to 0.8 M NH_4_Cl with optimum growth at 0.2 M. The major fatty acids are C_16 : 0_, C_14 : 0_ and C_18 : 0_. The following substrates are utilized: formate, carbohydrates (e.g. fructose, galactose and glucose) and aa (e.g. asparagine, betaine, casamino acids, histidine, serine and tryptone). Low growth was supported by cysteine, maltose, ribose and tryptophan. The following substrates were not utilized: acetate, acetoin, arabinose, benzoic acid, 1-butanol, 2,3-butandiol, cellobiose, citrate, dimethylamine, glycerol, ethylene glycol (5 mM, 0.3 g l^−1^), fumaric acid, lactose, lactate, leucine, malic acid, mannitol, mannose, methionine, methylamine, phenylalanine, proline, 1,2-propanediol, 2-propanol, pyruvate, raffinose, salicin, sorbitol, sucrose, syringate (2 mM, 0.39 g l^−1^), vanillic acid (3 mM, 0.50 g l^−1^) and xylose (2 mM, 0.30 g l^−1^). The major product is acetate. Nitrate and sulphate were not reduced in the presence of acetate.

The type strain is AMB_02^T^ (=DSM 110248^T^=JCM 39108^T^), and its DNA G+C content is 34.8%. The type strain was isolated from a lab-scale high-ammonia biogas system operated in Uppsala, Sweden. The GenBank accession number for its 16S rRNA gene sequence is MN365130.1, and its complete genome is deposited under accession number CP143259.1.

## Supplementary material

10.1099/ijsem.0.006773 Uncited Fig. S1.

## Abbreviations

AAI: average aa identity
ANI: average nt identity
BM: bicarbonate-buffered media
dDDH: digital DNA–DNA hybridization
MIS: Microbial Identification System
rGlyP: reductive glycine pathway
VFAs: volatile fatty acids
WLP: Wood–Ljungdahl pathway
