# Supplementary material for: Microaceticoccus formicicus gen. nov., sp. nov., an ammonia-tolerant formate-utilizing bacterium originating from a biogas process
Source: Int J Syst Evol Microbiol. 2025 May 8;75(5):006773. doi: 10.1099/ijsem.0.006773 (PMC12079837; doi:10.1099/ijsem.0.006773)
Supplement: Uncited Fig. S1. [file ijsem-75-06773-s001.pdf]

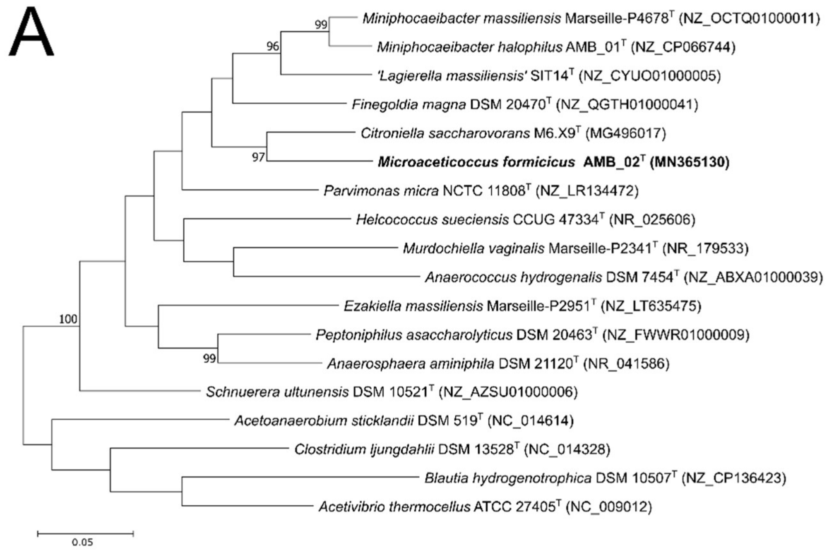

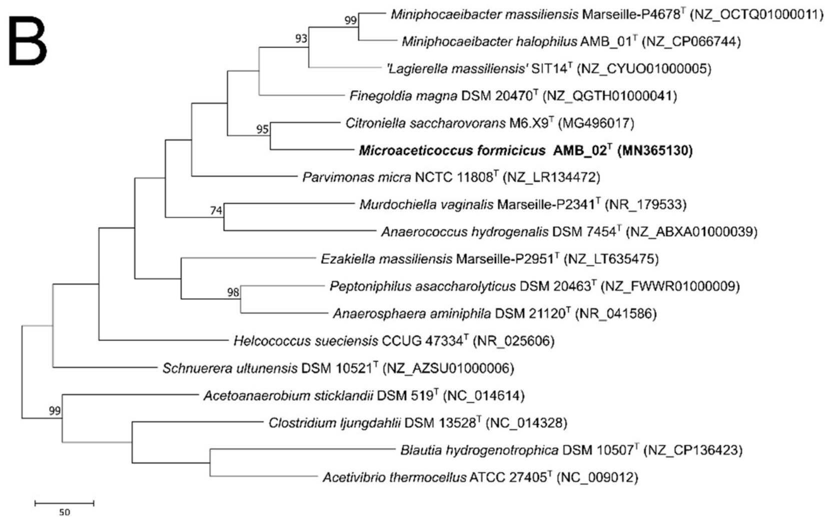

Fig. S1 Maximum-likelihood tree (A) and maximum-parsimony tree (B) based on 16S rRNA gene sequences of AMB_02^T and closely related species. Bootstrap values above 70% (based on 1000 iterations) are presented at the branch points, with bar 0.05 substitutions per nucleotide site (A) and bar with 50 nucleotide substitutions across branch length (B).

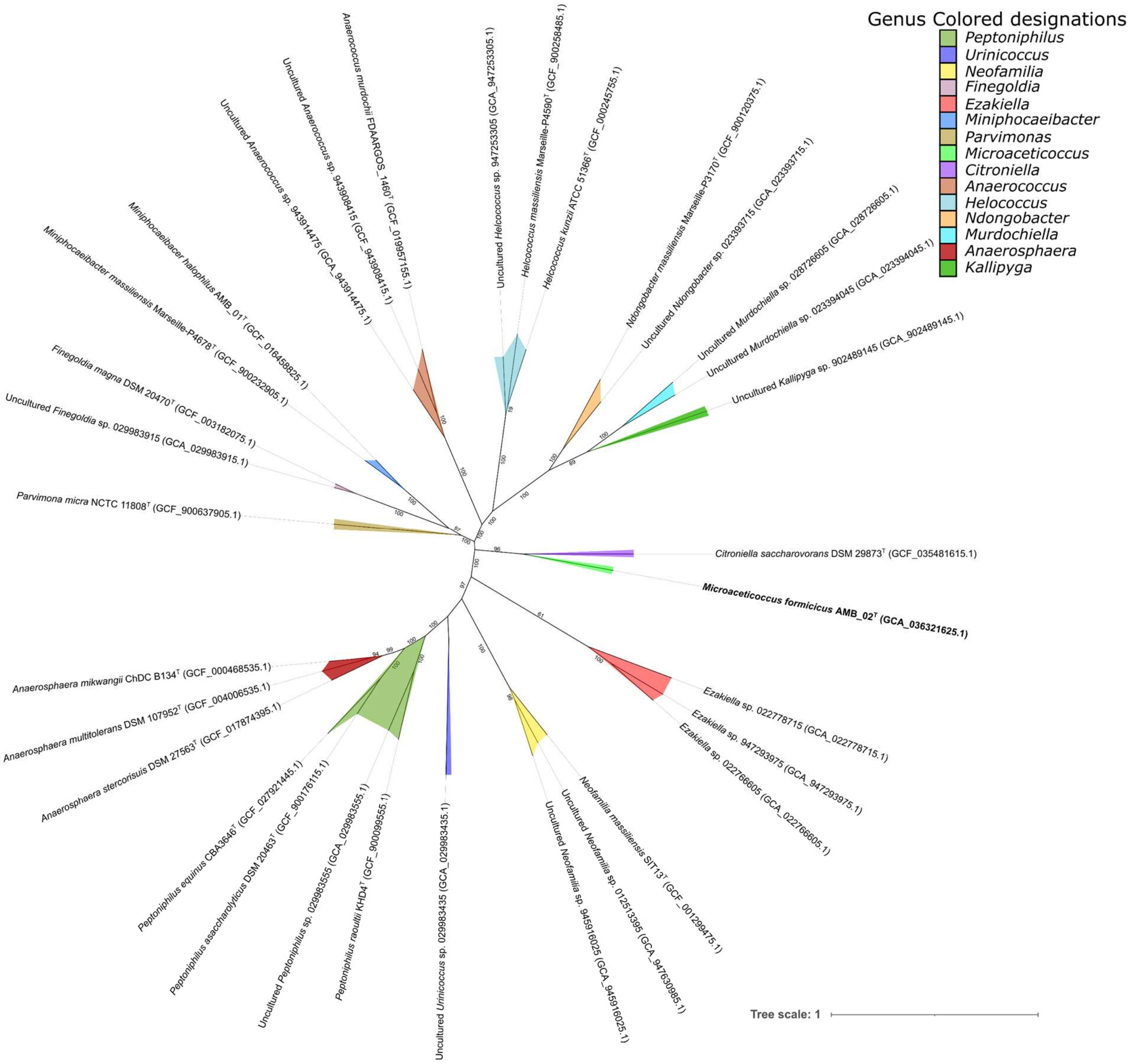

Fig. S2. Reconstructed GToTree species tree from sequence alignment of representatives from the *Peptoniphilaceae* family using 119 marker genes specific to the phylum *Bacillota* (formerly *Firmicutes*), to determine the tentative placement of strain AMB_02^{T}.

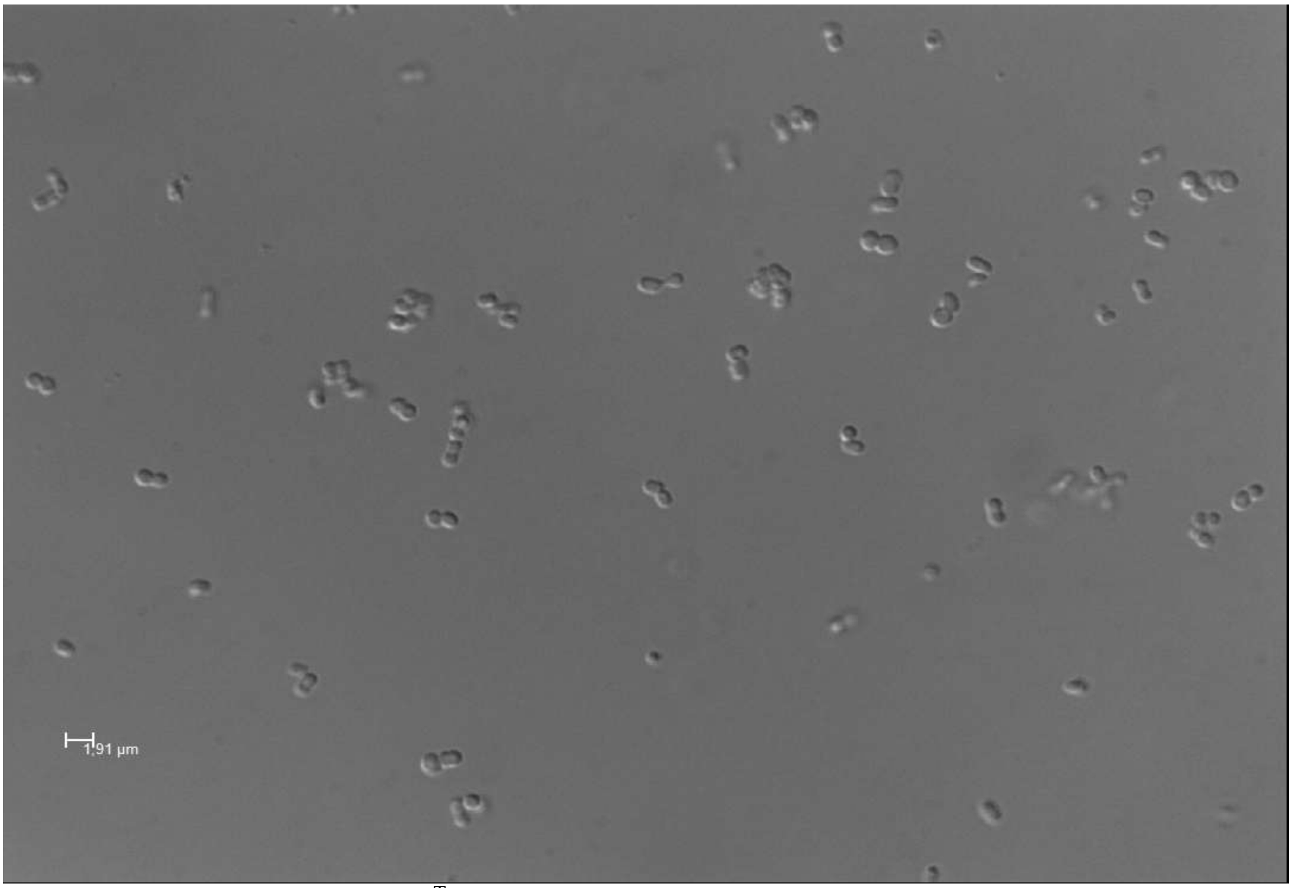

Fig. S3. Microscopy of AMB_02 [T]. The cells were spherical with a diameter about 0.5µm and had demonstrated motility.

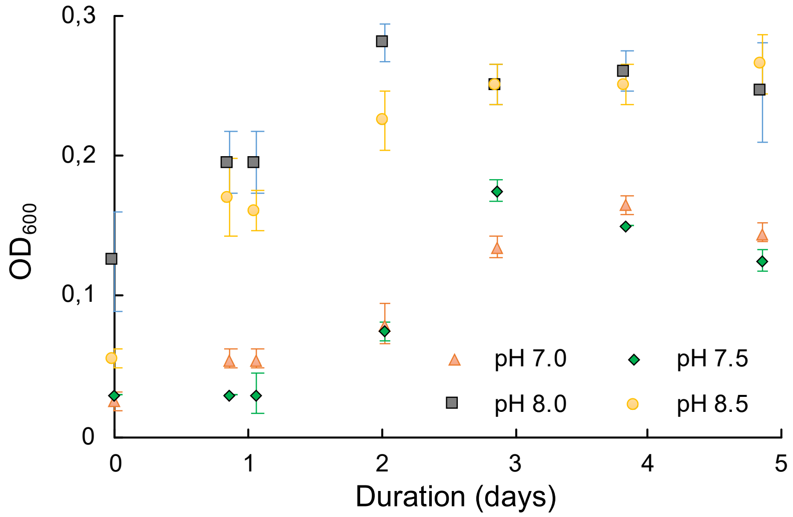

Fig. S4. Growth curves of strain AMB_02^T at different pH levels at 37ºC

Table S1 Detailed description of bicarbonate-buffered basal media (BM) prepared as described by Zehnder et al. 1980 [1] with modification as described in Westerholm et al. 2011 [2]. Additional instructions for pH adjustment with the addition of HCl and Na_2CO_3.

**Compounds**	**g/L**
KH_2PO_4	0.408
Na_2HPO_4	0.426
Resazurin $C_{12}H_6NaNO_3$	0.5 10^{-3}
$Na_2WO_4{\cdot}2H_2O$	32.9 10^{-6}
$Na_2SeO_3{\cdot}5H_2O$	26.3 10^{-6}
$NaHCO_3$	3.9
Yeast extract	0.2
**Trace elements solution**	
$FeCl_2$ $4H_2O$	2 10^{-3}
H_3BO_3	50 10^{-6}
$ZnCl_2$	50 10^{-6}
$CuCl_2$ $2H_2O$	38 10^{-6}
$MnCl_2$ $2H_2O$	41 10^{-6}
$(NH_4)_6Mo_7O_{24}$ $4H_2O$	50 10^{-6}
$AlCl_3$	50 10^{-6}
$CoCl_2{\cdot}6H_2O$	50 10^{-6}
$NiCl_2{\cdot}6H_2O$	80 10^{-6}
EDTA	
$C_{10}H_{14}N_2Na_2O_8{\cdot}2H_2O$	500 10^{-6}
Conc. HCl	1 10^{-6}
**Mineral salt solution**	
NH_4Cl	0.30
NaCl	0.30
$CaCl_2{\cdot}2H_2O$	0.11
$MgCl_2{\cdot}6H_2O$	0.10
**Reducing agent**	
Cystein-HCl	0.5
$Na_2S{\cdot}9H_2O$	0.24
**Vitamin solution**	
Pyridoxamine	2.5 10^{-4}
Nicotinic acid	1 10^{-4}
Nicotinamide	1 10^{-4}
DL-panthothenic acid	5 10^{-5}
Vitamin B_{12}	5 10^{-5}
p-aminobenzoic acid	5 10^{-5}
Pyridoxine hydrochloride	1 10^{-4}
Biotin	2 10^{-5}
Thioctic acid	5 10^{-5}
Folic acid	2 10^{-5}
Riboflavin	5 10^{-5}
Thiamine hydrochloride	1 10^{-4}

pH adjustment

To avoid pH drift during growth at different pH, the isolate was inoculated in media with the following modifications, KH_2PO_4 or Na_2HPO_4 was increased to 40 g/L for inoculation at low or high pH respectively. Minor pH adjustments were achieved by adding the required volume of HCl (1 M) for pH 6.0 to 7.0 or Na_2CO_3 (1 M) for pH 7.5 to 9.0 and the pH was verified with additional measurements.

Table S2 Medium 2 recipe adapted from McSweeney et al. 2005 [3]. Glucose, maltose, and cellobiose were omitted for the substrate test of AMB_02^T and *Citroniella saccharovorans* strain DSM 29873^T. Ingredients are in grams (g) unless specified otherwise and distilled H_2O is added to a final volume of 100mL.

**Ingredients**	**Grams (g)**
Casitone	1.0
Yeast extract	0.25
Sodium lactate (70% w/v)	1.0
Glucose	0.20
Maltose	0.20
Cellobiose	0.20
$NaHCO_3$	0.4
Solution 1	15 mL
Solution 2	15 mL
Resazurin	0.1 mg
**Reducing Agent**	
Cystein-HCl	0.05
**Solution 1**	
K_2HPO_4	6.0 g/L
**Solution 2**	
$CaCl_2{\cdot}H_2O$	1.6 g/L
KH_2PO_4	6.0 g/L
NaCl	12.0 g/L
$(NH_4)_2SO_4$	6.0 g/L
$MgSO_4{\cdot}7H_2O$	2.5 g/L

Table S3. Accession numbers of genomes and 16S rRNA gene sequences that were used for phylogenetic tree reconstruction.

Name	Strain[1]	Type Strain[2]	Genome Assembly Accession[3]	GenBank Accession	16S rRNA gene coordinates[4]	16S rRNA gene length (bp)
Acetoanaerobium sticklandii	DSM 519	Y	GCF_000196455.1[♦]	NC_014614	NC_014614.1:1096814-1098446[#]	1632
Blautia hydrogenotrophica	DSM 10507	Y	GCF_034356035.1[♦]	NZ_CP136423	NZ_CP136423.1:32445-33978[#]	1533
Miniphocaeibacter halophilus	AMB_01	Y	GCF_016458825.1[♦]	NZ_CP066744	NZ_CP066744.1:3895-5424[#]	1529
Clostridium ljungdahlii	DSM 13528	Y	GCF_000143685.1[♦]	NC_014328	NC_014328.1:9417-10926[#]	1509
Helcococcus sueciensis	DSM 17243[+] CCUG 47334*	Y	GCF_000423145.1[♦+]	NR_025606*	NR_025606.1[!]*	1512
Acetivibrio thermocellus	ATCC 27405	Y	GCF_000015865.1[♦]	NC_009012	NC_009012.1:3427586-3429113[#]	1527
'Lagierella massiliensis'	SIT14	Y	GCF_001407835.1[♦]	NZ_CYUO00000005	NZ_CYUO01000005.1:107890-109418[#]	1528
Miniphocaeibacter massiliensis	Marseille-P4678	Y	GCF_900232905.1[♦]	NZ_OCTQ01000011	NZ_OCTQ01000011.1:3750-5174[#]	1424
Parvimonas micra	NCTC 11808	Y	GCF_900637905.1[♦]	NZ_LR134472	NZ_LR134472.1:1150216-1151741[#]	1525
Schnuerera ultunensis	DSM 10521	Y	GCF_000511955.1[♦]	NZ_AZSU00000006	NZ_AZSU01000006.1:3503-5030[#]	1527
Anaerococcus hydrogenalis	DSM 7454	Y	GCF_000173355.1[♦]	NZ_ABXA01000039	NZ_ABXA01000039.1:533-2059[#]	1526
Anaerosphaera aminiphila	DSM 21120	Y	GCF_900129925.1[♦]	NR_041586	NR_041586.1[!]	1472
Ezakiella massiliensis	Marseille-P2951	Y	GCF_900120165.1[♦]	NZ_LT635475	NZ_LT635475.1:1740235-1741776[#]	1541
Finegoldia magna	DSM 20470	Y	GCF_003182075.1[♦]	NZ_QGTH01000041	NZ_QGTH01000041.1:3362-4883[#]	1521
Murdochiella vaginalis	Marseille-P2341	Y	GCF_900119705.1[♦]	NR_179533	NR_179533.1[!]	1488
Peptoniphilus asaccharolyticus	DSM 20463	Y	GCF_900176115.1[♦]	NZ_FWWR00000009	NZ_FWWR01000009.1:938432-939960[#]	1528
Citroniella saccharovorans	DSM 29873[+]	Y	GCF_035481615.1[♦+]	MG496017*	MG496017.1[!]*	1413

	M6.X9*					
***Microaceticoccus formicicus* AMB_02^{T}**	AMB_02	Y	GCA_036321625.1○	MN365130	MN365130.1!	1420

1 * - Corresponding strain to accession number for 16S rRNA gene, + - Corresponding strain to accession number for genome assembly

2 Y – yes, X – no

3 ♦ - RefSeq assembly, ○ - Genbank assembly

4 # - Extracted from assemblies, ! - Downloaded from NCBI

Table S4. Substrate growth characteristics comparison for 1, AMB_02^T; 2, *Citroniella saccharovorans* DSM 29873^T [4]; 3, *Finegoldia magna* DSM 20470^T [5]; 4, *Miniphocaeibacter halophilus* DSM 110247^T [6]; 5, *Parvimonas micra* ATCC 33270^T [7]. ++strong growth, +weak growth, -no reaction, ND no data. Information of medium used can be found in the corresponding references.

**Substrate**	**1!**	**2***	**3***	**4!**	**5***
Acetate	-	ND	ND	-	ND
Acetoin	-	ND	ND	-	ND
Arabinose	-	ND	ND	-	ND
Asparagine	++	++	ND	++	ND
Benzoic Acid	-	ND	ND	-	ND
Betaine	++	ND	ND	++	ND
1-Butanol	-	ND	ND	-	ND
2,3-Butandiol	-	ND	ND	-	ND
Casaamino Acid	++	ND	ND	-	ND
Cellobiose	-	++	ND	++	ND
Citrate	-	ND	ND	-	ND
L-Cysteine	+	ND	ND	++	++
Dimethylamine	-	ND	ND	-	ND
D-Galactose	++	+	ND	++	ND
D-Glucose	++	++	+	++	ND
Glycerol	-	ND	ND	++	ND
Ethanol	-	ND	ND	+	ND
Ethanolamin	-	ND	ND	-	ND
Ethylene glycol	-	ND	ND	-	ND
Formate	++	ND	ND	-	ND
Fructose	++	+	+	++	++
Fumaric acid	-	ND	ND	++	ND
Histidine	++	ND	+	-	ND
Lactose	-	ND	ND	-	ND
Lactate	-	ND	ND	-	ND
Leucine	-	ND	++	-	ND
Malic Acid	-	ND	ND	++	ND
Maltose	+	++	ND	++	ND
Mannitol	-	ND	ND	-	ND
Mannose	-	-	ND	++	ND
Methanol	-	ND	ND	-	ND
Methionine	-	ND	ND	-	ND
Methylamine	-	ND	ND	-	ND
Phenylalanine	-	ND	ND	-	ND
Proline	-	ND	-	-	ND
1,2-Propanediol	-	ND	ND	++	ND
2-Propanol	-	ND	ND	-	ND
Pyruvate	-	++	ND	++	ND
Raffinose	-	ND	ND	-	ND
Ribose	++	ND	ND	++	++
Salicin	-	ND	ND	-	ND
Serine	++	++	+	++	ND
Sorbitol	-	ND	ND	-	ND
Sucrose	-	ND	ND	-	ND
Syringate	-	ND	ND	-	ND
Tryptone	++	ND	ND	-	ND
Tryptophan	+	ND	ND	++	ND
Vanillic Acid	-	ND	ND	-	ND
Xylose	-	ND	ND	-	ND

! - substrate tests performed on bicarbonate-buffered basal media (BM).

*- for information on medium used please refer to the corresponding references.

Reference

1. **Zehnder AJB, Huser BA, Brock TD, Wuhrmann K**. Characterization of an acetate-decarboxylating, non-hydrogen-oxidizing methane bacterium. *Arch Microbiol* 1980;124:1–11.

2. **Westerholm M, Roos S, Schnürer A**. *Tepidanaerobacter acetatoxydans* sp. nov., an anaerobic, syntrophic acetate-oxidizing bacterium isolated from two ammonium-enriched mesophilic methanogenic processes. *Syst Appl Microbiol* 2011;34:260–266.

3. **McSweeney CS, Denman SE, Mackie RI**. Rumen bacteria. In: Makkar HPS, McSweeney CS (editors). *Methods in Gut Microbial Ecology for Ruminants*. Dordrecht: Springer Netherlands. pp. 23–37.

4. **Patel NB, Obregón-Tito AJ, Tito RY, Trujillo-Villaroel O, Marin-Reyes L, *et al.*** *Citroniella saccharovorans* gen. nov. sp. nov., a member of the family *Peptoniphilaceae* isolated from a human fecal sample from a coastal traditional community member. *International Journal of Systematic and Evolutionary Microbiology* 2019;69:1142–1148.

5. **Murdoch DA, Shah HN**. Reclassification of *Peptostreptococcus magnus* (Prevot 1933) Holdeman and Moore 1972 as *Finegoldia magna* comb. nov. and *Peptostreptococcus micros* (Prevot 1933) Smith 1957 as *Micromonas micros* comb. nov. *Anaerobe* 1999;5:555–559.

6. **Schnürer A, Singh A, Bi S, Qiao W, Westerholm M**. *Miniphocaeibacter halophilus* sp. nov., an ammonium-tolerant acetate-producing bacterium isolated from a biogas system. *International Journal of Systematic and Evolutionary Microbiology* 2022;72:005328.

7. **Tindall BJ, Euzéby JP**. Proposal of *Parvimonas* gen. nov. and *Quatrionicoccus* gen. nov. as replacements for the illegitimate, prokaryotic, generic names *Micromonas* Murdoch and Shah 2000 and *Quadricoccus* Maszenan et al. 2002, respectively. *International Journal of Systematic and Evolutionary Microbiology* 2006;56:2711–2713.
